# Expression of TLR-7, MyD88, NF-kB, and INF-α in B Lymphocytes of Mayan Women with Systemic Lupus Erythematosus in Mexico

**DOI:** 10.3389/fimmu.2016.00022

**Published:** 2016-02-02

**Authors:** Guillermo Valencia Pacheco, Irene B. Novelo Noh, Rubí M.-H. Velasco Cárdenas, Angélica V. Angulo Ramírez, Ricardo F. López Villanueva, Irma G. Quintal Ortiz, Ligia G. Alonso Salomón, Norma Pavía Ruz, Nubia A. Rivero Cárdenas

**Affiliations:** ^1^Laboratorio de Hematología, Centro de Investigaciones Regionales Dr. Hideyo Noguchi, Universidad Autónoma de Yucatán, Mérida, Mexico; ^2^Facultad de Química, Universidad Autónoma de Yucatán, Mérida, Mexico; ^3^Hospital General Dr. Agustín O’Horán, Mérida, Mexico; ^4^Hospital General Regional ISSSTE, Servicios de Salud de Yucatán (SSY), Mérida, México

**Keywords:** innate immunity, Toll-like receptor 7, Interferon-α, systemic lupus erythematosus, pathogenesis

## Abstract

**Background:**

Systemic lupus erythematosus (SLE) is a chronic inflammatory autoimmune disease involving multiple organs. It is currently accepted that several genetic, environmental, and hormonal factors are contributing to its development. Innate immunity may have a great influence in autoimmunity through Toll-like receptors. TLR-7 recognizing single-strand RNA has been involved in SLE. Its activation induces intracellular signal with attraction of MyD88 and NF-kBp65, leading to IFN-α synthesis which correlate with disease activity.

**Objective:**

To assess the expression of TLR-7, MyD88, and NF-kBp65 in B lymphocytes of Mayan women with SLE.

**Methods:**

One hundred patients with SLE and 100 healthy controls, all of them Mayan women, were included. TLR-7 was analyzed on B and T lymphocytes, and MyD88 and NF-kB only in B lymphocytes. Serum INF-α level was evaluated by ELISA.

**Results:**

Significant expression (*p* < 0.0001) of TLR-7 in B and T lymphocytes and serum IFN-α increased (*p* = 0.034) was observed in patients. MyD88 and NF-kBp65 were also increased in B lymphocytes of patients. TLR-7 and NF-kBp65 expression correlated, but no correlation with INF-α and disease activity was detected.

**Conclusion:**

Data support the role of TLR-7 and signal proteins in the pathogenesis of SLE in the Mayan population of Yucatán.

## Introduction

Systemic lupus erythematosus (SLE) is an autoimmune disease of connective tissue characterized by B lymphocytes hyperactivity and autoantibodies against nuclear self-antigens. The disease has a worldwide distribution and predominantly affects women. The SLE pathogenesis is yet unknown but several genetic, hormonal, and environmental factors are contributing to its development (1–4). The incidence of SLE patients varies according to the population studied (5, 6). Several studies have been conducted in patients from different populations (Asian, European, and American), but few in Mexican population. Mexico has an admixed Mestizo population with a genetic pool from the Amerindian and the Spanish (7). Mexican individuals with SLE appear to have a more severe disease and a lower age of onset than European women and a higher frequency of disease activity flares. Moreover, it has been reported that the prevalence of SLE in Yucatán (0.7%) is slightly higher than the national prevalence (0.6%) (8, 9), but immune studies have not been conducted in the Mayan population.

Studies have shown that abnormal stimulation of innate immunity may have a great influence on immunopathogenesis of SLE through Toll-like receptors (TLRs) (10, 11). Those are pattern-recognition receptors (PRR) that identify a broad range of pathogen-associated molecular patterns (PAMPs) (12, 13). So far, 11 human TLRs have been identified, and TLR-7 has been associated with SLE in both human and mouse models (14–19). This receptor is found on endosomes of several immune cells, mainly antigen-presenting cells, such as dendritic and B cells (20). The recognition and internalization, through the B cell receptor, of nuclear self-antigens released as a consequence of apoptosis in SLE patients, can activate TLR-7 in endosomes of B lymphocytes supporting its role in the production of autoantibodies (21–24). RNA-containing complexes must access the interior of the plasmacytoid dendritic cells (pDCs), through the Fc receptors, thus providing a route of entry for RNA to reach TLR-7, with the resulting INF-α production (25, 26). INF-α influences the development, progression, and pathogenesis of SLE (27–30). As a result of TLR-7 ligation, INF-α enhances TLR-7 signaling in pDCs forming a positive feedback loop (31, 32).

The TLR-7 ligation induce signal transduction via the myeloid differentiation primary-response protein 88 (MyD88), a common adaptor protein, which interacts with IRAK1/4 (Interleukin-1 receptor-associated kinase 1/4) and TRAF6 (TNF receptor-associated factor 6) to form the MyD88/IRAK1/IRAK4/TRAF6 complex. Subsequently, IRAK1 and TRAF6 dissociate from the receptor complex and interact with kinases IKKB (IκB kinases) resulting in the activation of NF-kB (nuclear factor kappa-light-chain-enhancer of activated B cells), permitting the expression of genes of proinflammatory cytokine and chemokines (33, 34). On the other hand, the transcription factor IRF-7 (Interferon regulatory factor 7) can bind to the MyD88/IRAK1/IRAK4 complex, and its activation is dependent upon TLR-7 requiring the TRAF3 (TNF receptor-associated factor 3) protein, which joins IRAK1 and IKKα kinases to produce IFN-α (34).

Previously, the copy number variation (CNV) of *TLR-7* gene in 80 Mayan women with SLE was analyzed in our laboratory. We found that 10% of patients had more than two copies of the *TLR-7* gene. These data suggest that increased CNV of the TLR7 gene may be a risk factor in this population (35). However, the expression of TLR-7 and signaling proteins has not been analyzed in B lymphocytes of our patients. Our aim was to assess the TLR-7, MyD88, and NF-kBp65 expression in B cells of Mayan women with SLE and to compare them to healthy controls. Protein expressions were correlated with serum INF-α and disease activity.

## Materials and Methods

### SLE Patients

One hundred SLE women of Mayan origin were recruited at the Rheumatology outpatient of the Agustin O’Horán and ISSSTE Regional Hospital, Yucatán. Diagnosis was established according to the American College of Rheumatology (ACR) criteria (36), and disease activity was evaluated by SLEDAI score (37). One hundred healthy women of the same origin were studied as controls. All selected subjects included in the study gave their informed consent, according to the Declaration of Helsinki. The study was approved by the Research Ethics Committee of the Agustin O’Horán Hospital of Yucatán (CIE-008-1-11). All women gave 15 ml of venous peripheral blood in one collection.

### Cell Isolation

Ten milliliters of venous peripheral blood were collected in heparinized tubes. Peripheral blood mononuclear cells (PBMC) were isolated from each subject, either patient and control, by gradient centrifugation on Ficoll-Hypaque (NycoPrep 1.077, Axis-Shield PoC AS, Oslo, Norway), and the cell viability and concentration was determined by staining with trypan blue and counted in a Neubauer chamber. Cells were adjusted to a concentration of 1 × 10^6^ cells/ml in complete medium [RPMI 1640 supplemented with 10% fetal bovine serum (FBS), 100 U/ml penicillin, 100 μg/ml streptomycin, and 2.0 mM l-glutamine].

### TLR-7, MyD88, and NF-kBp65 Expression

Isolated PBMC (1 × 10^6^ cells/tube) were first incubated with surface monoclonal antibodies against CD19 (Clone 1D3) and CD4 (Clone RPA-T4) conjugated with fluorescein isothiocyanate (FITC) and allophycocyanin (APC), respectively (IMGENEX, San Diego, CA, USA), in darkness at 4°C for 30 min. The cells were then fixed and permeabilized using the IC-Flow kit (IMGENEX, 10083K Cat, San Diego, CA, USA), and incubated with monoclonal antibodies against TLR-7 (Clone 4G6), MyD88 (Clone 4D6), and NF-kBp65 (Clone 2J10D7) conjugated with phycoerythrin (PE), in darkness at 4°C for 30 min. Mouse IgG conjugated with FITC, PE, and APC were included as isotype controls (all from IMGENEX, San Diego, CA, USA). The cells were finally washed and assessed by flow cytometry. A total of 10,000 cells were analyzed in the flow cytometer (FACScalibur, BD Biosciences Corp., San Jose, CA, USA) using the Cell Quest software. The lymphocytes population was identified using the forward scatter (FSC) versus side scatter (SSC) distribution. The percentages of CD19^+^ B and CD4^+^ cells expressing TLR-7 and CD19^+^ B cells expressing MyD88 and NF-kBp65 were assessed. The relative fluorescence intensity (rFI) of TLR-7, MyD88, and NF-kBp65 was calculated based on the mean fluorescence intensity of the sample (MFIs) compared with isotype control (MFIc), using the formula: rFI = MFIs − MFIc/MFIc.

### Interferon-Alpha

Five milliliters of venous peripheral blood (without anticoagulant) were selected from each patient and control subject to obtain serum. Serum levels of IFNα in were determined by VeriKine Human IFN-alpha Serum Sample ELISA kit, following the directions of the supplier (PBL Assay Science Piscataway, NJ, USA). The kit quantitates human IFNα using a sandwich immunoassay, with an anti-secondary antibody conjugated to horseradish peroxidase (HRP) and tetramethylbenzidine (TMB) as substrate. The detection range of 12.5–1000 pg/ml was calculated using a standard curve. Each standard, blank, and sample test was run in duplicate. The absorbance was determined at 450 nm, using a microplate reader (BIOTEK Instrument, Inc., VT, USA).

### Statistical Analysis

Wilcoxon matched-pairs signed rank test was used to assess the significance of any difference in values of TLR-7, MyD88, NF-kBp65 expression and IFNα serum, among SLE patients and control subjects (*p* < 0.05). Correlation analysis was done using the Pearson correlation coefficient. In all comparisons, the level of significance was *p* < 0.05, using the Graph Pad Prism 5 software.

## Results

### Characteristics of SLE Patients

All patients were under treatment, 54% of them had active disease determined by SLEDAI (>4), and most residents of the Merida city (54%), the rest from the surrounding Yucatán state. The average age of patients was 39.73 years with different times of evolution (Table 1).

**Table 1:** **Characteristics of SLE patients**.

Features	SLE patients (%) or mean ± SD (range)
Number	100
Age (year)	39.73 ± 12.8973 (18–69)
Disease duration (year)	8.11 ± 6.9101 (0.01–29)
SLEDAI	
Non-active (SLEDAI > 4)	46%
Active (SLEDAI < 4)	54%
Locality in the Yucatán state	
Mérida	54%
Progreso	4%
Motul	4%
Izamal	2%
Dzidzantún	2%
Other in Yucatán state	34%
Treatment	
Prednisone	49%^a^
Mycophenolic acid	10%^a^
Azathioprine	32%^a^
Hydroxychloroquine	13%^a^
Deflazacort	24%^a^
Methotrexate	14%^a^

*SLEDAI, Systemic Lupus Erythematosus Disease Activity Index*.

*^a^Percentage of patients receiving the drug in combination with other one*.

### Expression of TLR-7, MyD88, and NF-kBp65

**Figure 1:**
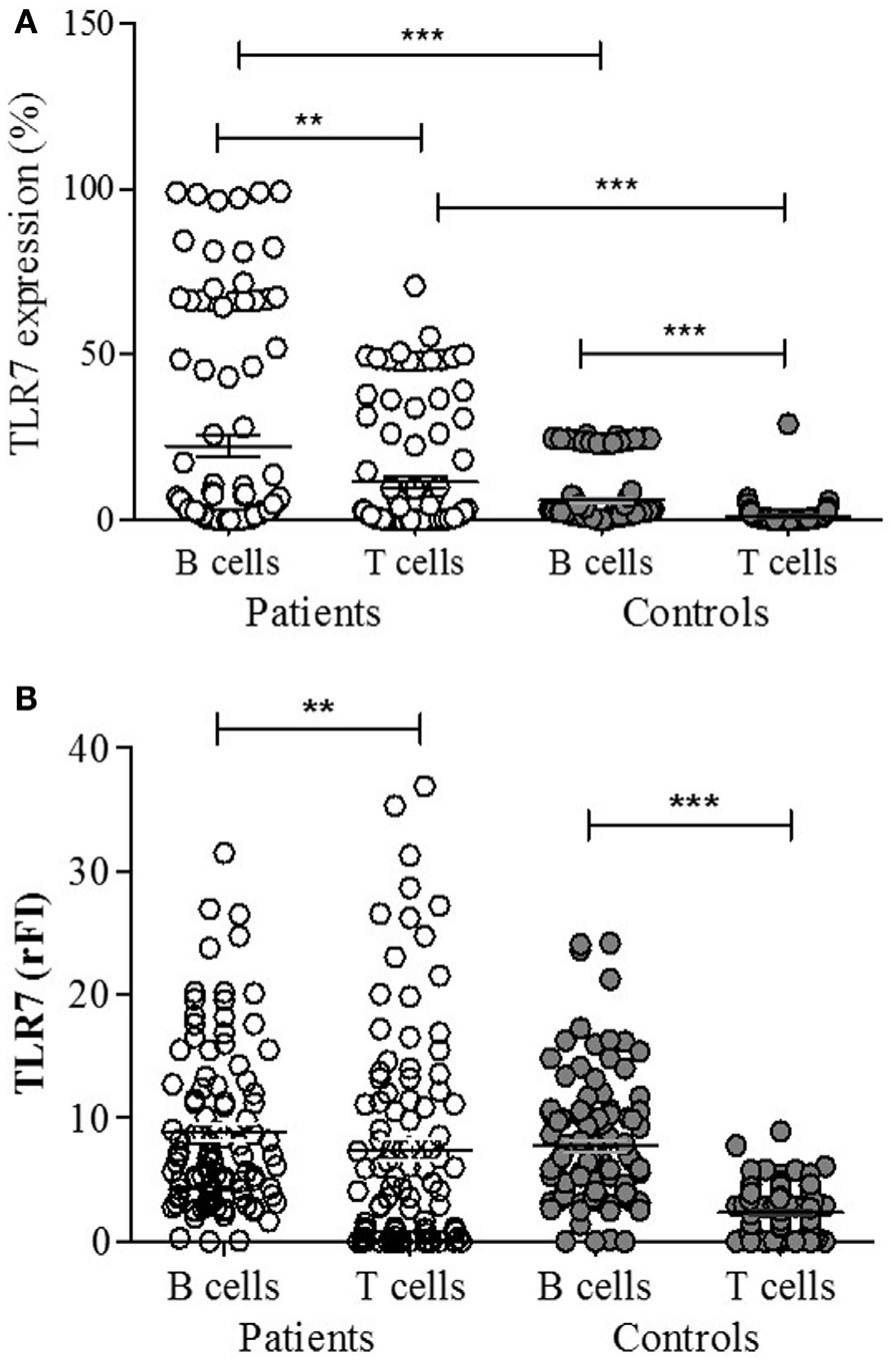
**TLR-7 expression in B (CD19^+^) and T (CD4^+^) lymphocytes of SLE patients (*n* = 100) and control subjects (*n* = 100), analyzed by flow cytometry**. Results expressed as the percentage **(A)** and relative fluorescence intensity (rFI) **(B)** of TLR-7 are presented in scatter plots and mean with SEM. Wilcoxon matched-pairs signed rank test was used to assess the difference of expression among SLE patients and control subject. **p = 0.0004, ***p < 0.0001.

**Figure 2:**
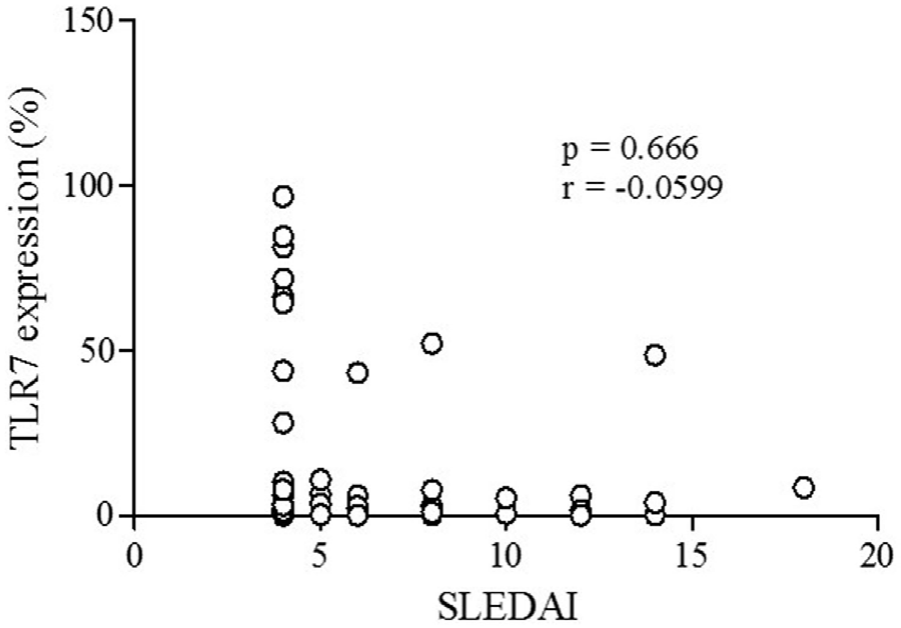
**Correlation analysis of TLR-7 expression (%) with active disease (SLEDAI > 4) in SLE patients**. Results are presented in scatter plots. Pearson correlation test was used to assess correlation, *r* = Pearson correlation coefficient; *p* < 0.05.

A representative figure of the analysis by flow cytometry, as described in Section “Materials and Methods,” is shown in Figure S1 in Supplementary Material. A higher percentage of CD19^+^ B lymphocytes expressing TLR-7 were found in patients compared to controls (*p* < 0.0001) (Figure 1A). The rFI of TLR-7 was significantly higher in B lymphocytes of patients, but no difference was found between patients and controls (*p* = 0.1882) (Figure 1B). Furthermore, significant expression of TLR-7 was found in CD4^+^ T lymphocytes of patients with respect to controls (*p* < 0.0001), and the rFI of TLR-7 was higher in patients. Additionally, no correlation between TLR-7 expression in CD19^+^ B lymphocytes and active disease (SLEDAI > 4) was observed (Figure 2).

**Figure 3:**
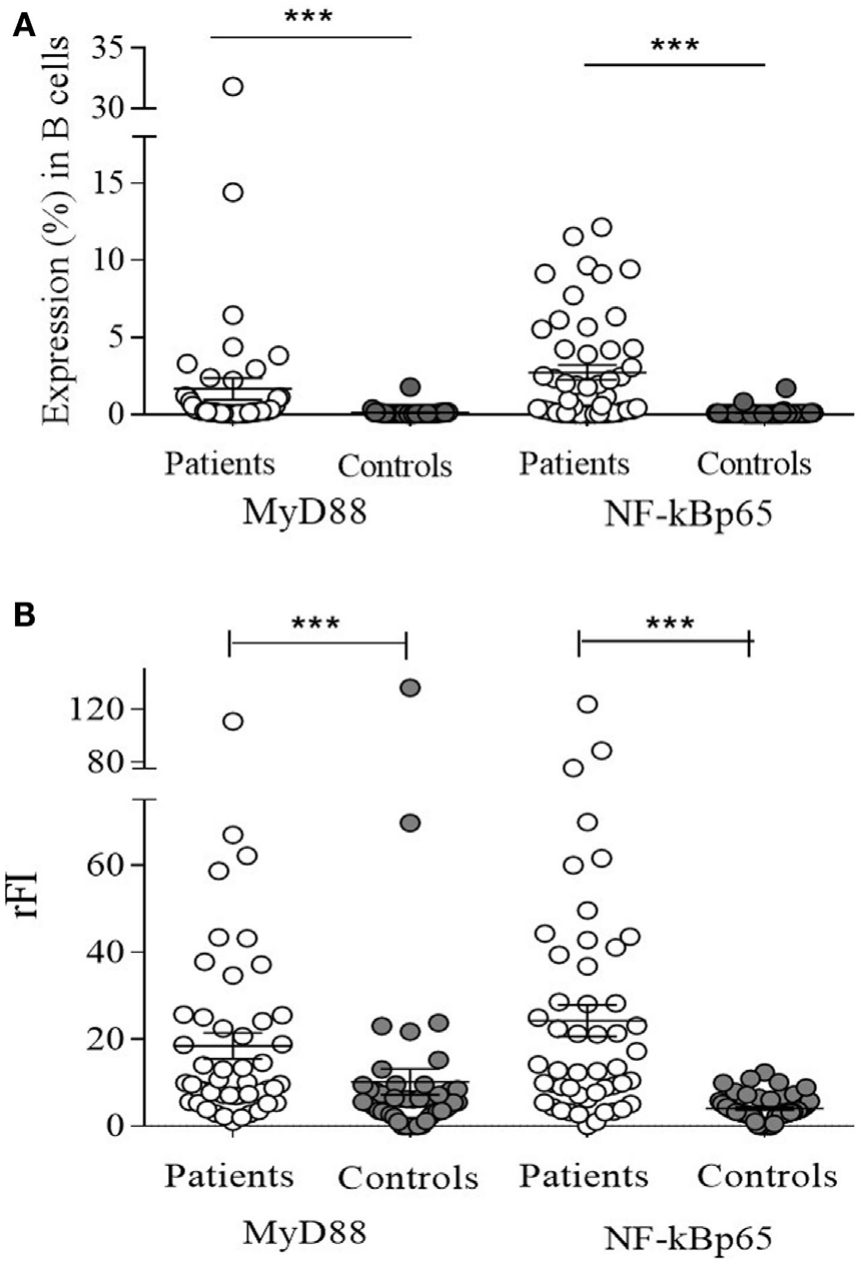
**MyD88 and NF-kBp65 expression in B lymphocytes of SLE patients (*n* = 50) and control subjects (*n* = 50), analyzed by flow cytometry**. Results expressed as the percentage **(A)** and relative fluorescence intensity (rFI) **(B)** of MyD88 and XF-kBp65 is presented in scatter plots and mean with SEM. Wilcoxon matched-pairs signed rank test was used to assess the difference of expression among SLE patients and control subject. ***p < 0.0001.

**Figure 4:**
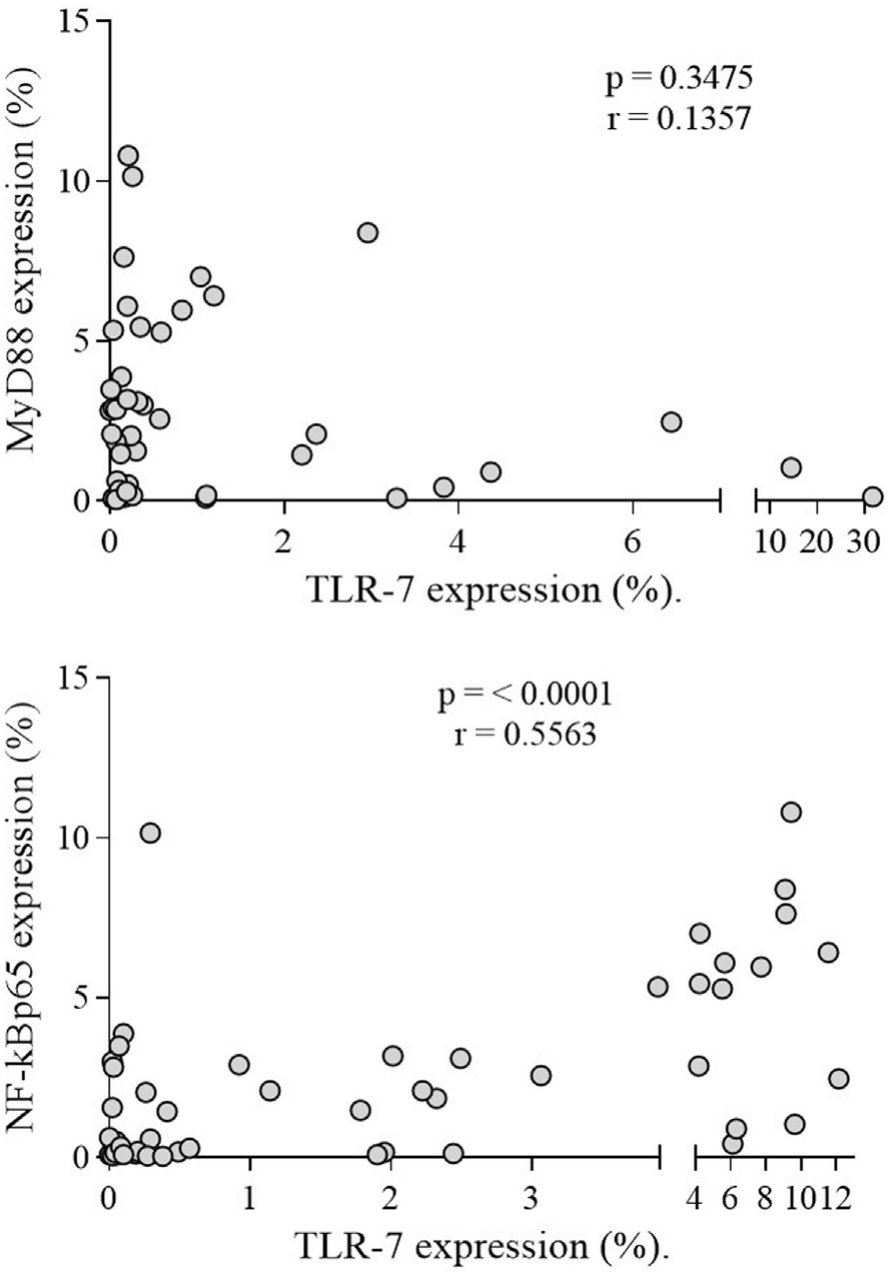
**Correlation analysis of TLR-7 with MyDSS and NF-kBp65 expression in SLE patients**. Results are presented in scatter plots. Pearson correlation test was used to assess correlation, *r* = Pearson correlation coefficient; *p* < 0.05.

Regarding MyD88 and NF-kBp65, both were expressed more in CD19^+^ B lymphocytes of patients (*n* = 50) than of controls (*n* = 50) (*p* < 0.0001) (Figure 3A). Only NF-kBp65 correlated with TLR-7 expression in B lymphocytes of patients (Figure 4). The rFI of both proteins was significantly increased in CD19^+^ B lymphocytes from patients (*p* < 0.0001) (Figure 3B), but no correlation with TLR-7 expression was observed (Figure S2 in Supplementary Material). No correlation with active disease (SLEDAI > 4) was observed with both proteins.

### Serum IFN-α

**Figure 5:**
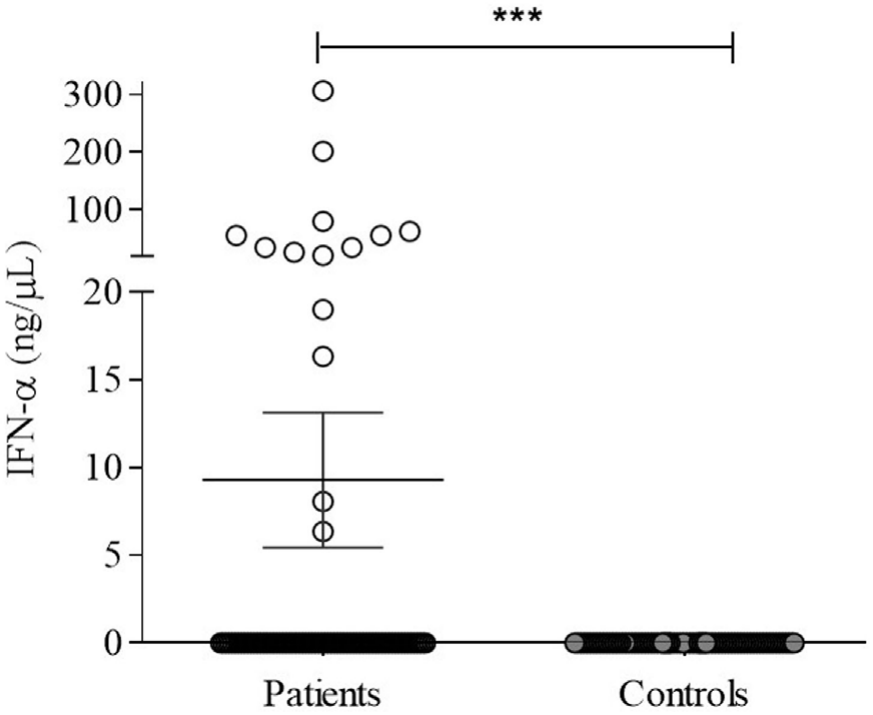
**Serum IFN-α in SLE patients (*n* = 100) and controls subjects (*n* = 100) were detected by human IFN-α ELISA, as described in Section “Materials and Methods.”** Results expressed as nanograms per picoliter of IFN-α are presented in scatter plots and mean with SEM. Wilcoxon matched-pairs signed rank test was used to assess the difference among SLE patients and control subject. ***p = 0.0001.

**Figure 6:**
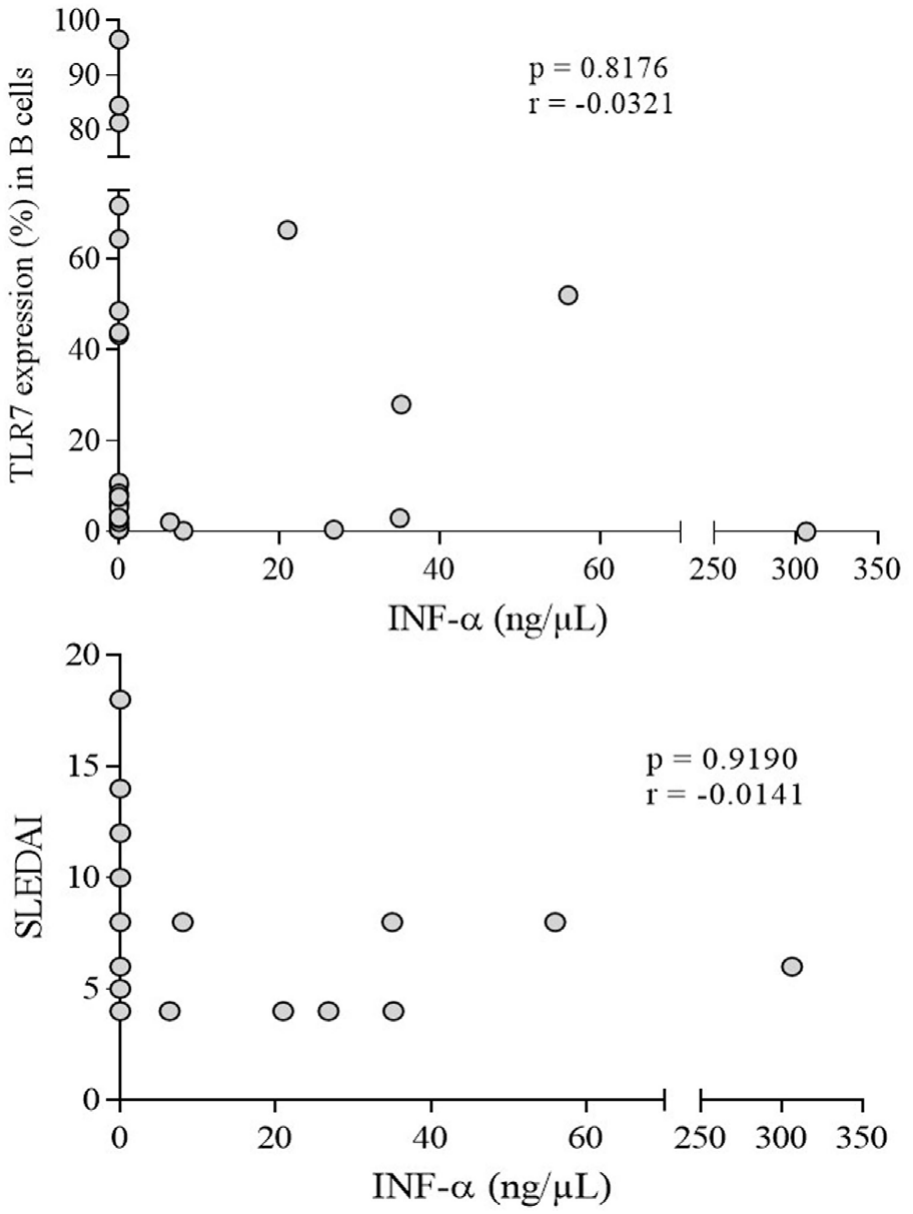
**Correlation analysis of serum IFN-α with TLR-7 expression (%) and active disease (SLEDAI > 4) in SLE patients**. Results are presented in scatter plots. Pearson correlation test was used to assess correlation, *r* = Pearson correlation coefficient; *p* < 0.05.

IFN-α was variable in patients (7.6 ± 3.54 ng/μl) but significantly higher (*p* = 0.0001) compared to undetectable levels in controls (Figure 5). However, no correlation with TLR-7 expression and disease activity (SLEDAI > 4) was observed (Figure 6).

## Discussion

This research represented the first report on the expression of TLR-7 and signal proteins MyD88 and NF-kBp65 in B lymphocytes of Mayan women with SLE. We observed significant increase of TLR-7 expression in B lymphocytes of SLE patients compared to control subjects, which was consistent with previous studies, even with different analysis procedures (38–41). The rFI of TLR7 also was increased in B cells from patients. This suggested that overexpression of TLR-7 in B lymphocytes may be a common characteristic of SLE patients since TLR-7 are the main source of pathological antibodies for the disease. Moreover, correlation between the TLR-7 expression and disease severity has been reported in SLE patients; however, we did not find correlation probably due to therapy they already received. Further study is needed to clarify this discrepancy.

Although a high percentage of B lymphocytes expressing TLR-7 were observed in patients, different subtypes of CD4^+^ T cells also express TLRs (42). The TLR-7 has been reported on CD4^+^CD25^+^ T regulatory (Treg) cells, and TLR-7 activation increase their suppressor function by suppressing autoreactive lymphocytes, but defects in their number and function may contribute to pathogenesis of SLE (43–45). It has been reported IL-17 secretion by human CD4 T cells stimulated with TLR-7 agonist, suggesting that TLR-7 ligation generates proinflammatory cytokines that induces Th17 differentiation and establishes a link between TLR-7 interaction and Th17 cell differentiation (46). An imbalanced Th17/Treg ratio favoring Th17 cells has been reported in SLE patients (47). We observed higher expression of TLR-7 in CD4^+^ T cells in SLE patients, but subtypes of Treg and Th17 cells were not identified. Further researches are needed to strengthen the role of TLR-7 on these cells as a mechanism of action in autoimmunity.

Few studies have evaluated the expression of MyD88 in cells of SLE patients. Nakano et al. assessed the mRNA of MyD88 in Chinese patients B lymphocytes, and found no significant difference with controls (48). Chen et al. studied the role of the TLR-7 signaling pathway in the pathogenesis of adult-onset Still’s disease (AOSD) and SLE, finding increased levels of mRNA of MyD88, TRAF6, IRAK-4, and IFN-α in mononuclear cell of SLE patients, which correlated with disease activity (49). Data suggested that overexpression of MyD88-dependent signaling molecules may be a pathogenesis mechanism in SLE. We found significant expression levels of MyD88 in B lymphocytes of patients by flow cytometry, but no correlation with TLR-7 expression and disease activity was found, suggesting that activation of TLR-7 signaling pathway in our SLE patients appeared to be unaffected by the disease activity. Further studies are needed to establish whether MyD88 expression levels correlate with its mRNA or are influenced by the activation of other intracellular receptors that share the molecule, and if the treatment received by patients has any impact on MyD88.

The NF-kBp65 is an inducible transcription factor that controls genes involved in inflammatory responses and play an important role in B lymphocytes maintenance (50–52). Genetic associations have been found between genes involved in NF-kBp65 signaling pathway in Chinese SLE patients, highlighting the role of NF-kBp65 in autoimmunity (53). We found significantly higher expression levels of NF-kBp65 in B lymphocytes from SLE patients consistent with those reported (53), suggesting its constitutive activation in B cells of patients; however, no correlation with TLR-7 expression and disease activity was observed. Data support its role in the mechanisms of autoimmunity, but further studies are needed to identify which receptor induces NF-kBp65 activation, promoting the survival of autoreactive B lymphocytes in SLE patients, despite treatment received.

A hallmark of SLE is the elevated levels of INF-α in serum. Approximately 50% of patients have been shown to have dysregulated expression of genes involved in the INF pathway, which correlates with disease activity (54, 55). High levels of INF-α were detected in our patients, but no correlation with disease activity was found. It is important to note that although 54% of patients had active disease, and all of them showed variable levels of serum INF-α, inactive patients showed low and undetectable levels. This variability is likely due to the effect of the drugs they received. Studies in murine models have reported the inhibitory effect of chloroquine and corticosteroids on the immune response. Acidification of lysosomes and function of TLR-7 and TLR-9 are inhibited by hydroxychloroquine, and its activation and release of cytokines is suppressed by prednisone (56, 57). In this regard, 54% of our patients were receiving prednisone in combination with other drugs, suggesting that the combined effect of drugs may modify the inflammatory response and inhibit the synthesis of cytokines, including INF-α. Longitudinal studies are needed to determine the effect of therapy on the synthesis of INF-α and disease activity.

## Conclusion

Our results show increased expression of TLR-7, MyD88, and NF-kBp65 in B lymphocytes from Mayan women, which supports its role in the pathogenesis of SLE in this ethnic population of southeast of Mexico.

## Author Contributions

Conceived and designed the experiments: GP. Analyzed the data: CO, IN, and RC. Contributed to the writing of the manuscript: AR and RV. All authors reviewed and approved the final manuscript.

## Conflict of Interest Statement

The authors declare that the research was conducted in the absence of any commercial or financial relationships that could be construed as a potential conflict of interest.
