# Supplementary figures and images for: Expression of TLR-7, MyD88, NF-kB, and INF-α in B Lymphocytes of Mayan Women with Systemic Lupus Erythematosus in Mexico
Source: Front Immunol. 2016 Feb 2;7:22. doi: 10.3389/fimmu.2016.00022 (PMC4735402; doi:10.3389/fimmu.2016.00022)

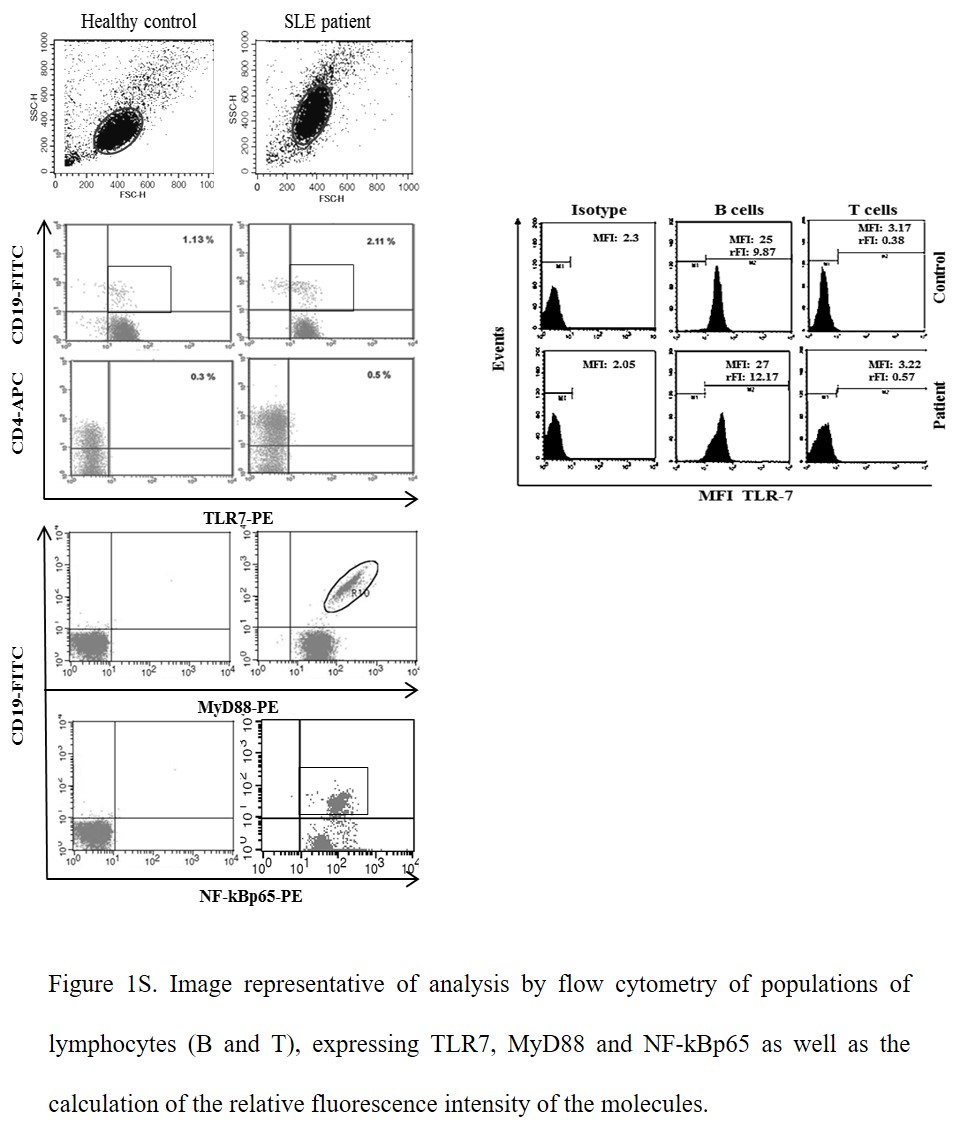

Supplement: Supplementary file 1 [file Image_1.JPG]

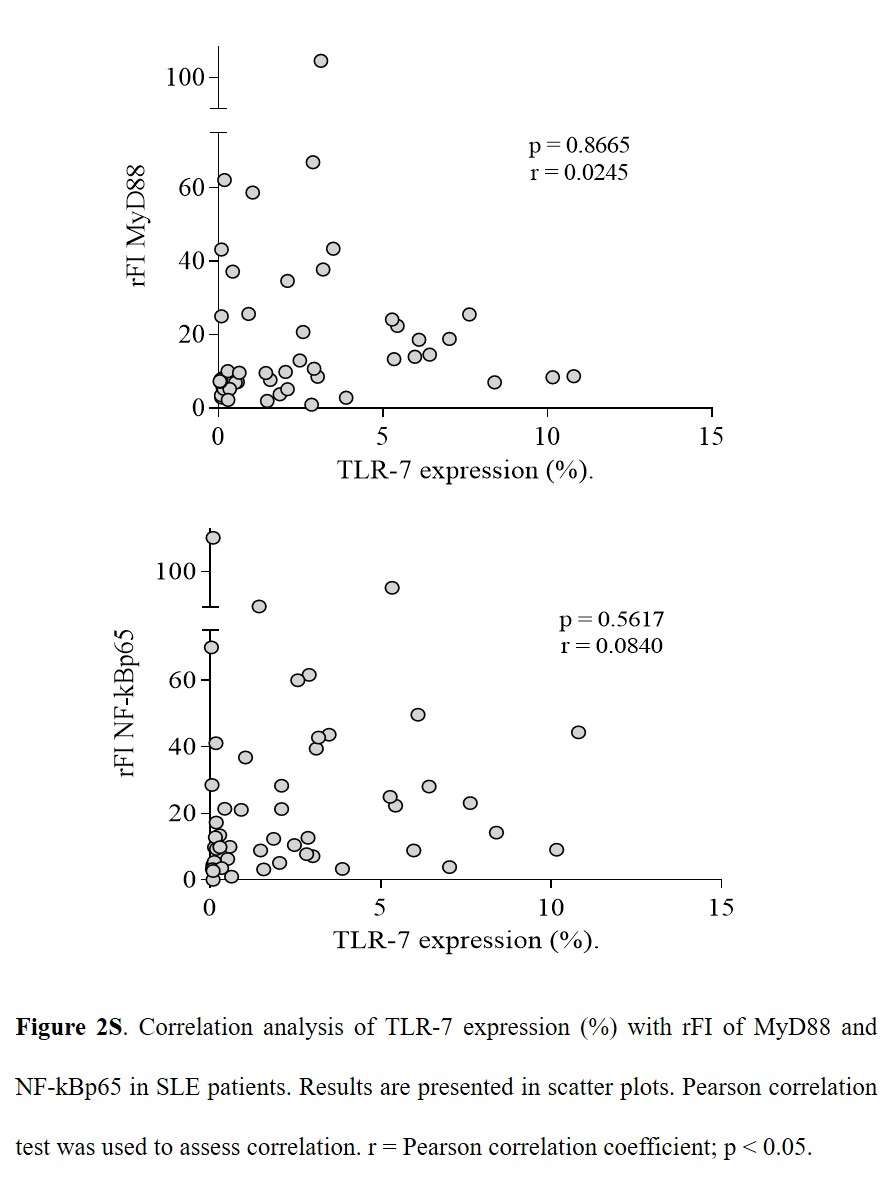

Supplement: Supplementary file 2 [file Image_2.JPG]
